# Hepatocellular Carcinoma–Related Mortality Trends Associated With Infective Versus Non-Infective Etiologies in ≥ 35 Years Age Group

**DOI:** 10.14740/gr2129

**Published:** 2026-06-16

**Authors:** Jay Kakadiya, Yash Nasit, Manthan Vaghani, Smit Bhalala, Areeba Fatima, Jayasree Rajapandian, Kush Varsadiya, Zunirah Ahmed

**Affiliations:** a Department of Internal Medicine, Government Medical College, Surat, India; b Department of Internal Medicine, Dow Medical College, Karachi, Pakistan; c Department of Internal Medicine, The University of Alabama at Birmingham, Montgomery, AL, USA; d Department of Internal Medicine, Shree M.P. Shah Medical College, Jamnagar, India; e Division of Gastroenterology and Hepatology, Kansas University of Medical Center, Kansas City, KS, USA

**Keywords:** Hepatocellular carcinoma, Hepatitis B, Hepatitis C, Metabolic dysfunction–associated fatty liver disease, Alcohol-associated liver disease, CDC Wonder

## Abstract

**Background:**

Hepatocellular carcinoma (HCC) is a leading cause of cancer-related mortality worldwide, with evolving etiologic patterns. While viral hepatitis has historically been the predominant cause, non-infective etiologies such as alcohol-associated liver disease and metabolic dysfunction–associated fatty liver disease (MAFLD) are increasingly driving the disease burden. We evaluated long-term trends in HCC-related mortality in the United States by etiology and demographic subgroups.

**Methods:**

Mortality data from 1999 to 2020 were obtained from the Centers for Disease Control and Prevention’s Wide-ranging ONline Data for Epidemiologic Research (CDC WONDER) database. HCC-related deaths were classified as infective (viral hepatitis–associated) or non-infective (liver disease and metabolic disorder) using International Statistical Classification of Diseases and Related Health Problems, 10th Revision (ICD-10) codes. Age-adjusted mortality rates (AAMRs) were extracted using CDC WONDER. Joinpoint regression was used to assess temporal trends and estimate annual percent change (APC) and average annual percent change (AAPC). Analyses were stratified by sex, age, race/ethnicity, geographic region, and urbanization.

**Results:**

Overall HCC-related mortality increased substantially from 1999 to 2020, driven predominantly by non-infective etiologies. Infective AAMRs rose from 1999 to 2013 followed by a significant decline, whereas non-infective AAMRs increased persistently from 1999 to 2020. Mortality rates were consistently higher in males than females. Infective mortality was highest among Asians, while non-infective mortality disproportionately affected Hispanic and American Indian populations. Western US states exhibited the highest mortality rates, and non-infective etiologies accounted for most deaths among individuals aged ≥ 65 years.

**Conclusions:**

HCC-related mortality in the United States has increased over the past two decades, largely driven by non-infective etiologies. Targeted public health strategies addressing metabolic risk factors, alcohol use, and demographic disparities are urgently needed to curb this growing burden.

## Introduction

Hepatocellular carcinoma (HCC) is one of the most common primary malignant neoplasms of the liver and represents a major contributor to cancer-related morbidity and mortality worldwide. Incidence and mortality associated with HCC increased by 26% and 25% respectively from 2010 to 2021 [1]. In 2020 alone, an estimated 906,000 new diagnoses and 782,000 deaths were attributed to HCC [2], and projections suggest that this figure will approach 1.4 million annually by 2040 [2]. This highlights the requirement of effective prevention and control strategies by understanding the drivers of this rise.

HCC arises from both infectious and non-infectious causes, each affecting the liver through distinct mechanism. Hepatitis B virus (HBV) and hepatitis C virus (HCV) account for 33% and 21% of HCC cases diagnosed worldwide, respectively [3]. Integration of the HBV genome into the host genome, causing genomic instability, is the primary pathway of oncogenesis [4]. HCV, an RNA virus, does not integrate into the host genome; instead, it promotes carcinogenesis through chronic inflammation, immune-mediated hepatocyte injury, and progressive fibrosis leading to cirrhosis [5]. Non-infectious causes including liver disease and metabolic dysfunction–associated fatty liver disease (MAFLD) account for nearly 45% of HCC-related deaths [3]. Autoimmune hepatitis (AIH) is another important non-infectious cause that predisposes patients to cirrhosis and subsequently increases the risk of HCC, and the reported incidence of HCC is 1.007% (10.07 per 1,000 person-years), compared with only 0.114% (1.14 per 1,000 person-years) in non-cirrhotic AIH cohorts [6]. Across non-infectious etiologies, the shared pathogenic pathway involves chronic liver injury, steatosis, persistent inflammation, and progressive fibrosis, culminating in cirrhosis [7, 8].

Current trends suggest that non-infectious causes will overtake infectious causes as the dominant drivers of HCC by 2040–2050 [2]. Addressing this shift requires a comprehensive, multifaceted approach that includes vaccination, antiviral treatments for infective causes, as well as lifestyle modification, early detection, and focused screening efforts for metabolic risk factors.

We utilized mortality data from the Centers for Disease Control and Prevention’s Wide-ranging ONline Data for Epidemiologic Research (CDC WONDER) [9] to analyze trends of HCC-related mortality in the United States over the last 20 years (1999–2020). By comparing HCC-related mortality rates associated with infectious versus non-infectious factors and assessing disparities across sex, race, age groups, and geographic regions, this study aims to provide insights that can guide public health policies, enhance preventive strategies, and mitigate the growing impact of HCC on US populations.

## Materials and Methods

### Data source and study designs

We utilized data from CDC WONDER database [9], a comprehensive and nationally representative source of mortality statistics in the United States. Our analysis covered the period from 1999 to 2020, specifically focusing on HCC-related mortality in ≥ 35 years of age of population in the United States.

We employed the International Statistical Classification of Diseases and Related Health Problems, 10th Revision (ICD-10) [10] coding system to perform diagnostic coding. Etiologic classification of HCC-related mortality (ICD-10 CM: C22.0) was operationalized through concurrent ICD-10 coding on death certificates, distinguishing between infective and non-infective factors. Infective causes included all deaths associated with viral hepatitis. Non-infective causes were defined as deaths associated with chronic liver disease and metabolic syndrome. To maintain epidemiologic relevance, analyses were restricted to adults aged ≥ 35 years, corresponding to the minimum age at which HCC-related mortality becomes statistically significant in US cohorts. Detailed summary of ICD-10 codes utilized in this study is given in Supplementary Material 1 (gr.elmerpub.com).

### Ethical statements

The data used in this study were obtained from publicly available, government-maintained datasets containing fully de-identified information. Because all data were aggregated and lacked personal identifiers, this study was exempt from Institutional Review Board (IRB) approval. The analysis adhered to the principles outlined in the Strengthening the Reporting of Observational Studies in Epidemiology (STROBE) [11] guidelines for transparent reporting of observational research.

### Data extraction

Mortality and population-level data for this study were retrieved from the publicly available CDC WONDER database. Extracted variables included annual death counts and population size by year, census region, place of death, and demographic characteristics such as age, gender, and race/ethnicity. Place of death was classified across multiple healthcare settings including inpatient, outpatient, home, hospice, and nursing home or long-term care facility.

Race/ethnicity was categorized as Hispanic and non-Hispanic further subclassified by American Indian, African-American, Asian, and White. County-level urbanization status was defined using the National Center for Health Statistics (NCHS) [12] 2013 Urban–Rural Classification Scheme for Counties, which classifies all US counties into six categories including large central metropolitan, large fringe metropolitan, medium metropolitan, small metropolitan, micropolitan, and noncore. This classification is based on the 2010 US Census [13] and the February 2013 Office of Management and Budget (OMB) [14] delineation of metropolitan and micropolitan statistical areas and is widely applied in national health surveillance research.

### Statistical analysis

Annual and subgroup-specific age-adjusted mortality rates (AAMRs) were calculated using direct standardization to the 2000 US standard population. Analyses were stratified by etiology, sex, age group, race/ethnicity, and US region. Temporal trends in AAMRs from 1999 through 2020 were evaluated using the Joinpoint Regression Program (Version 5.0.2, National Cancer Institute, Bethesda, MD, USA) [15], which employs log-linear regression models to identify statistically significant changes in trends over time. The optimal number and location of joinpoints were determined using the weighted Bayesian Information Criterion (BIC) [16]. A maximum of two joinpoints per model was permitted. Annual percent change (APC) and average annual percent change (AAPC) were estimated for each segment with 95% confidence intervals (CIs). Trends were categorized as increasing or decreasing based on whether the slope differed significantly from zero, assessed through two-tailed testing. Statistical significance was established at a threshold of P < 0.05. Only trends with 95% CIs not including zero were considered statistically significant. Descriptive summaries, group comparisons, and visualizations were generated using Joinpoint Regression Program and Microsoft Excel [17].

## Results

Mortality rates from HCC demonstrated substantial variation across etiologies, demographic profile, geographic regions, and social determinants. This study highlights trends and disparities in the most common causes of HCC-related mortality. Majority deaths occurred either in inpatient medical facilities or at home. Hospice facilities and long-term care settings accounted for a moderate proportion of deaths, while outpatient or emergency departments represented comparatively smaller shares (Table 1).

**Table 1:** HCC-Related Mortality Stratified by Common Etiologies

	Non-infectious	MAFLD	ALD	Infectious	Hepatitis C	Hepatitis B
Race (AAMR)						
American Indian	2.8	1.5	0.6	1.4	1.3	0.1
Asian	2.7	1.5	0.1	2.6	1.1	1.5
African American	2.2	1.3	0.3	2.1	1.9	0.3
White	1.5	0.9	0.2	0.9	0.8	0.1
Hispanic origin (AAMR)						
Hispanic	3.1	1.9	0.6	1.5	1.5	0.1
Not Hispanic	1.5	0.9	0.2	1	0.9	0.2
Place of death (death counts)						
Medical Facility - Inpatient	26,816	15,178	3,009	15,100	12,927	2,485
Medical Facility - Outpatient/ER	1,288	798	179	928	785	149
Decedent’s home	23,385	15,011	3,669	15,734	14,075	1,854
Hospice facility	5,662	3,376	1,321	4,825	4,460	457
Nursing home/long-term care	5,026	3,020	817	3,227	2,928	365

AAMR: age-adjusted mortality rate; ALD: alcohol-associated liver disease; ER: emergency room; HCC: hepatocellular carcinoma; MAFLD: metabolic dysfunction–associated liver disease.

### Trends in HCC-related mortality stratified by sex

**Figure 1:**
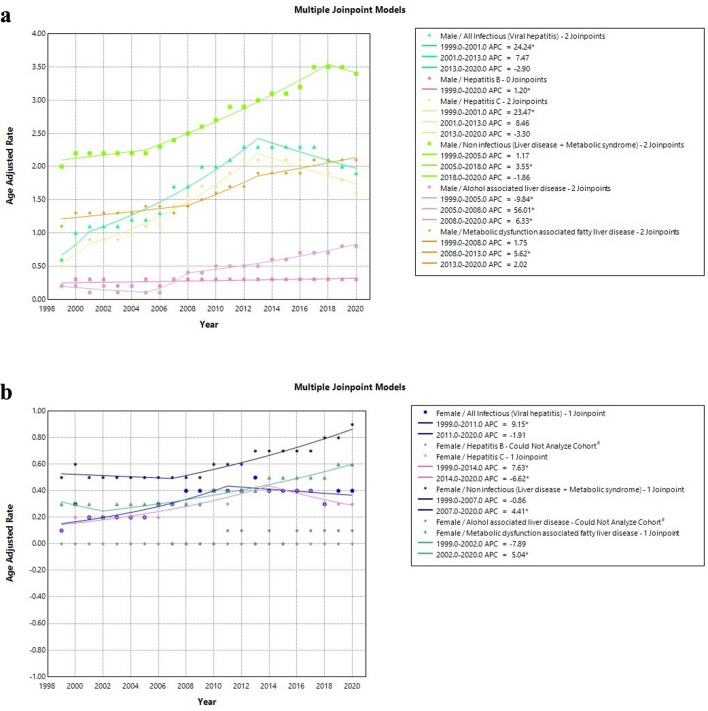
HCC-related mortality trends, 1999–2020, stratified by etiologies: (a) male; (b) female. *Statistically significant (P < 0.05). ^#^Could not able to analyze due to zero AAMR at some point of time. AAMR: age-adjusted mortality rate; HCC: hepatocellular carcinoma.

Mortality associated with HCC showed substantial variation between males and females across infectious and non-infectious etiologies. Trends in AAMR from 1999 to 2020 were evaluated using jointpoint regression, with result displayed graphically in Figure 1.

#### Male trends

Among males, overall AAMR associated with infectious etiologies increased from 0.6 in 1999 to 1.9 in 2020 with an AAPC of 5.34 (95% CI: 4.06–6.47, P < 0.01). Notably, AAMR raised continuously through 2013 (APC: 24.24, P < 0.01 (1999–2001); APC: 7.47, P = 0.10 (2001–2013)), followed by a decline from 2013 to 2020 (APC: −2.89, P = 0.11).

Mortality related to non-infectious etiologies also raised from 2.0 in 1999 to 3.4 in 2020 with an AAPC of 2.34 (95% CI: 2.05–2.61, P < 0.01). Unlike infectious etiologies, non-infectious etiology–related rates gradually increased from 1999 to 2005 (APC: 1.17, P = 0.18), accelerated between 2005 to 2018 (APC: 3.55, P < 0.01), and then decreased till 2020 (APC: −1.86, P = 0.44).

Subgroup analysis demonstrated most significant increase in alcohol-associated liver disease (ALD) AAMR with AAPC of 7.14 (95% CI: 4.73–9.14, P < 0.01). Detailed trend pattern with identified joinpoints is illustrated in Figure 1a.

#### Female trends

Among females, overall AAMR related to infectious etiologies rose from 0.1 in 1999 to 0.4 in 2020 with an AAPC of 4.27 (95% CI: 2.25–6.49, P < 0.01). Mortality rates raised continuously through 2011 (APC: 9.15, P < 0.01), followed by a decline through 2020 (APC: −1.91, P = 0.39).

Non-infectious etiology related rates also raised from 0.5 in 1999 to 0.9 in 2020 with an AAPC of 2.37 (95% CI: 1.81–2.95, P < 0.01). Notably, mortality rates gradually decreased from 1999 to 2007 (APC: −0.86, P = 0.28), followed by an increase through 2020 (APC: 4.41, P < 0.01).

Subgroup analysis showed a comparable rise in MAFLD-related mortality with an AAPC of 3.09 (95% CI: 2.23–4.62, P < 0.01) and HCV-related mortality with an AAPC of 3.35 (95% CI: 1.49–4.90, P < 0.01). Detailed trend analysis with identified joinpoints is shown in Figure 1b.

### HCC-related AAMR stratified by race and ethnicity

Marked racial and ethnic disparities were observed in AAMR. Infectious etiology–related mortality was highest among Asian cohort (2.6) followed by African American (2.1) and American Indian (1.4) individuals. Subgroup pattern was similar for hepatitis B; however, hepatitis C–related mortality is most experienced by African American (1.9).

For non-infectious etiologies, American Indians showed the highest AAMR (2.8), followed by Asians (2.7) and African Americans (2.2). Subgroup analysis demonstrated a similar pattern for MAFLD mortality. White population exhibited the lowest AAMR across nearly all etiologies except for ALD-related mortality.

Ethnic patterns mirrored these findings. Individuals of Hispanic or Latino origin showed higher mortality across all causes, except hepatitis B compared with non-Hispanic individuals. Detailed race- and ethnicity-specific mortality distributions are summarized in Table 1.

### Geographic and urbanization disparities

Geographically, non-infectious HCC-related mortality consistently exceeded infectious mortality across the United States. Western and Southwestern states—particularly Texas, New Mexico, California, and Hawaii—showed higher mortality levels for both categories. In contrast, several Midwestern and Southeastern states displayed comparatively lower mortality.

**Figure 2:**
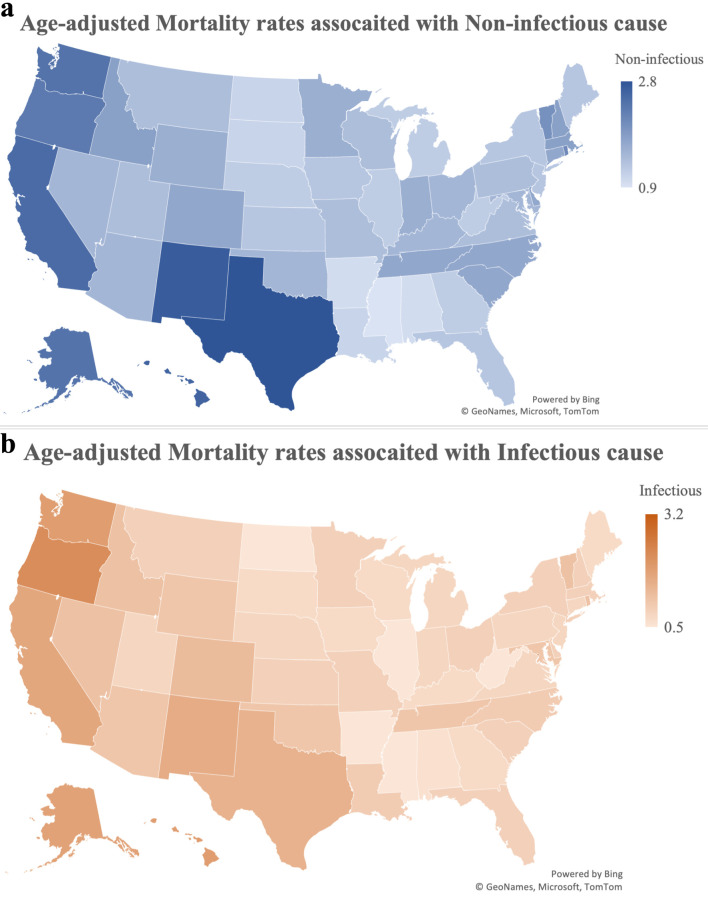
State-wise representation of HCC-related AAMR, 1999–2020: (a) non-infectious vs. (b) infectious. AAMR: age-adjusted mortality rate; HCC: hepatocellular carcinoma.

The highest infectious AAMR was observed in the District of Columbia (3.2) followed by Oregon (2.2), Washington (1.9), Hawaii (1.9), Alaska (1.8), and California (1.7) predominantly involving Western states. Lower mortality values were observed in states such as West Virginia (0.5), North Dakota (0.5), Mississippi (0.5), Arkansas (0.5), and Illinois (0.5). A similar pattern was observed in non-infectious mortality as well with the highest AAMR in Texas (2.8) followed by New Mexico (2.7), Hawaii (2.5), and California (2.4) involving western states. Central and southeastern states such as Mississippi (0.9), Alabama (1.0), Arkansas (1.0), Georgia (1.2), North Dakota (1.1), and South Dakota (1.1) showed comparatively lower rates. Map illustration of geographic variation of infectious and non-infectious etiologies–related AAMR across United States is demonstrated in Figure 2a and b, respectively.

**Figure 3:**
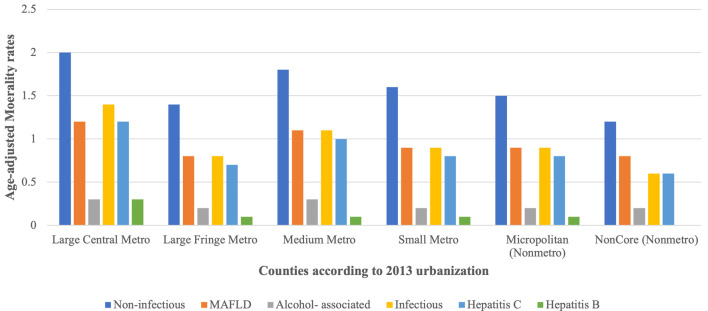
HCC-related AAMR stratified by urbanization. AAMR: age-adjusted mortality rate; HCC: hepatocellular carcinoma.

Urbanization analyses revealed that large central metropolitan areas experienced the highest mortality rates for all etiologies, with non-infectious etiologies (2.0) vs. infectious (1.4). However, noncore rural areas had the lowest rates, with higher rates in non-infectious (1.2) than infectious (0.6). Medium and small metro areas showed intermediate patterns. Detailed distribution is illustrated in Figure 3.

### HCC-related crude death rate (CDR) stratified by age group

**Figure 4:**
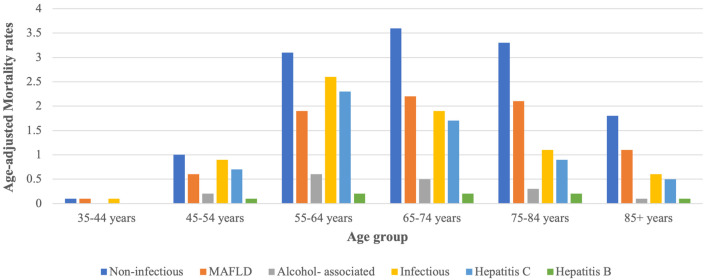
Crude death rates for HCC-related mortality stratified by age group (35+). HCC: hepatocellular carcinoma.

CDR increased markedly with age followed by decline across etiologies. Infectious CDR increased from 0.1 in 35–44 years age group to a peak of 2.6 in the 55–64 group and then gradually decreased in older ages. Subgroup analysis revealed that hepatitis B mortality remained consistently low (0.1–0.2), while hepatitis C mortality peaked at 2.3 in the 55–64 group before decreasing, representing the major contributor to infectious mortality (Fig. 4).

Non-infectious mortality showed a right skewed distribution, rising from 0.1 in 35–44 age group to a peak of 3.6 in 65–74 years age group, before declining slightly in the oldest group. Subgroup analysis reflected similar age-group wise mortality pattern in MAFLD but ALD had the highest mortality at 0.6 in 55–64 years group with consistently higher rates in MAFLD etiologies (Fig. 4).

As displayed in Figure 4, non-infectious etiologies accounted for most deaths among individuals aged ≥ 65, whereas infectious causes contributed a relatively larger proportion of mortality in younger adults.

## Discussion

Our study demonstrates a consistent increase in HCC-related mortality associated with non-infective etiologies in the United States from 1999 to 2020. In contrast, mortality associated with infective causes showed a more modest increase in AAMR over this period with significant decline between 2014 and 2020. Males had higher AAMRs as compared to females. Significant racial and ethnic disparities were also identified. Among infective causes, Asians had the highest mortality, whereas among non-infective causes, American Indian had the highest mortality rates. Moreover, residents of the western United States demonstrated the highest AAMR, while mid-west had the lowest.

These findings are consistent with the broader epidemiological shift documented in prior literature, a transition from infectious to non-infectious drivers of HCC-related mortality [18–21]. A large study reported a decline of hepatitis B–related deaths and relatively stable hepatitis C–related deaths [18], which is partially consistent with our results. It is attributed to improved therapeutic efficacy of anti-viral therapy, widespread immunization programs, and enhanced screening strategies that have reduced the burden of infective etiologies.

In contrast, the rise in non-infective mortality mirrors the increasing prevalence of MAFLD, driven by parallel increase in obesity and type 2 diabetes mellitus in the context of increasingly sedentary lifestyle [19]. Prior studies have reported substantial rise in incidence and death rate due to HCC associated with MAFLD in recent years, which aligns with our result [20, 22]. However, we observed higher mortality rates in the United States compared to global estimates from previous studies [22], which may reflect the comparatively greater prevalence of obesity in the US population [23].

Sex-based differences in HCC-related mortality were consistent with prior reports [3, 22, 24]. Males demonstrated higher AAMRs than females across all age groups, with the disparity becoming more pronounced with advancing age [25]. Several biological and behavioral factors may underlie these differences. The higher burden in males is thought to be driven by sex-specific gene expression patterns, androgen-mediated stimulation of hepatocarcinogens, and a relatively attenuated immune response. In addition, males have higher exposure of established risk factors, including alcohol consumption, smoking, and obesity, all of which contribute to liver disease progression and malignancy [26, 27]. Conversely, estrogen is believed to exert a protective effect against hepatic steatosis, fibrosis, and carcinogenesis, thereby reducing HCC susceptibility in females [26–28].

Racial and ethnic disparities were also evident in HCC-related mortality. We observed a disproportionately higher non-infective mortality burden among Hispanic and American Indian populations, suggesting an increasing impact of MAFLD and ALD in this population. This finding is partially consistent with prior reports indicating elevated incidence rates of HCC among Hispanics [3, 29, 30]. Additionally, Hispanic American Indian showed notably higher non-infective mortality. However, variations across studies have been reported, likely reflecting differences in demographic composition, regional risk factor prevalence, and healthcare access among study populations [25, 31, 32].

Beyond epidemiological trends, the nature of HCC is significantly different between infective and non-infective etiologies, with important prognostic implications. Viral hepatitis-related HCC associated with less advanced fibrosis compared to HCC associated with MAFLD or ALD, where cirrhosis is present universally at the time of diagnosis [33]. Different nature of HCC has direct clinical consequences. Patients with viral HCC are more likely to have preserved hepatic reserve, making them better candidates for curative therapies including surgical resection or liver transplantation. A recent cohort study comparing median survival between viral and non-viral HCC reported significantly better outcomes in patients with viral etiology. Furthermore, viral hepatitis-related HCC presents at a younger age and more often detected through surveillance programs, facilitating early diagnosis and advancement of antiviral therapies contributing to more favorable outcomes. In contrast, MAFLD and ALD often occur in older patients with multiple comorbidities, further limiting comparable preventive and therapeutic infrastructure. These differences underscore integrated policies emphasizing early detection, lifestyle modification, alcohol regulation, metabolic disease management, and equitable access to liver health screening. This requires not only prevention of new cases but also optimization of treatment option to the underlying liver disease.

This study has several limitations that should be acknowledged. First, the analysis relied solely on publicly available open-access mortality data from the CDC WONDER database, which is based on death certificate information and therefore subject to potential bias of misclassification or underreporting of causes of death. Second, the database does not provide individual-level clinical data, limiting our ability to adjust for potential confounders such as comorbidities, treatment history, socioeconomic status, or behavioral risk factors. Additionally, classification of etiologies as infective or non-infective was based on underlying/multiple cause-of-death coding, which may not be entirely capture mixed or multifactorial complex cases of HCC. Temporal changes in diagnostic accuracy, coding practices, and reporting standards over the study period may also have influenced observed trends. Finally, the CDC WONDER database does not include detailed information on disease stage and histologic subtype, preventing a more detailed assessment of survival differences or treatment outcomes. Despite these limitations, the large, nationally representative dataset provides valuable insight into long-term mortality trends and demographic disparities in HCC across the United States.

In summary, our analysis showed that HCC-related mortality in the United States increased between 1999 and 2020, with non-infective etiologies accounting for a growing proportion of deaths. While mortality associated with viral hepatitis demonstrated relatively modest changes, deaths linked to MAFLD or ALD rose consistently across most demographic and geographic subgroups. Persistent disparities by sex, age, race/ethnicity, and region were observed. These findings suggest an ongoing shift in the etiologic landscape of HCC and highlight the importance of strengthening prevention, early detection, and risk-factor modification strategies to address the evolving burden of HCC.

## Supplementary Material

Suppl 1 ICD-10 codes utilized for study.

## Data Availability

The data supporting the findings of this study are available from the corresponding author upon reasonable request.

## Abbreviations

AAMR: age-adjusted mortality rate
ALD: alcohol-associated liver disease
APC: annual percent change
AAPC: average annual percent change
CDC: Centers for Disease Control and Prevention
CDR: crude death rate
CI: confidence interval
HBV: hepatitis B virus
HCC: hepatocellular carcinoma
HCV: hepatitis C virus
ICD-10: International Statistical Classification of Diseases and Related Health Problems, Tenth Revision
IRB: Institutional Review Board
MAFLD: metabolic dysfunction–associated fatty liver disease
NCHS: National Center for Health Statistics
OMB: Office of Management and Budget
STROBE: Strengthening the Reporting of Observational Studies in Epidemiology
WONDER: Wide-ranging ONline Data for Epidemiologic Research
