# Supplementary material for: Hepatocellular Carcinoma–Related Mortality Trends Associated With Infective Versus Non-Infective Etiologies in ≥ 35 Years Age Group
Source: Gastroenterology Res. 2026 Jun 16;19(3):127–35. doi: 10.14740/gr2129 (PMC13278711; doi:10.14740/gr2129)
Supplement: Suppl 1 — ICD-10 codes utilized for study. [file gr-19-03-127-s001.docx]

| **Category** | **Variable** | **ICD-10-CM Codes** |
| --- | --- | --- |
| **Malignancy** | Hepatocellular carcinoma (HCC) | C22.0 (Liver cell carcinoma) |
| **Infective etiologies** | Viral hepatitis (overall) | B15 – B19 |
|  | Hepatitis B | B16.0 (Acute hepatitis B with delta-agent (coinfection) with hepatic coma)  B16.1 (Acute hepatitis B with delta-agent (coinfection) without hepatic coma)  B16.2 (Acute hepatitis B without delta-agent with hepatic coma)  B16.9 (Acute hepatitis B without delta-agent and without hepatic coma)  B17.0 (Acute delta-(super)infection of hepatitis B carrier)  B18.0 (Chronic viral hepatitis B with delta-agent)  B18.1 (Chronic viral hepatitis B without delta-agent) |
|  | Hepatitis C | B17.1 (Acute hepatitis C)  B18.2 (Chronic viral hepatitis C) |
| **Non-infective etiologies** | Liver disease and Metabolic dysfunction associated disorders (overall) | K70 – K76; E70 – E89 |
|  | Alcohol-associated liver disease | K70.0 (Alcoholic fatty liver)  K70.1 (Alcoholic hepatitis)  K70.2 (Alcoholic fibrosis and sclerosis of liver)  K70.3 (Alcoholic cirrhosis of liver)  K70.4 (Alcoholic hepatic failure)  K70.9 (Alcoholic liver disease, unspecified) |
|  | Metabolic dysfunction associated fatty liver disease (MAFLD) | K74.0, K74.1, K74.2, K74.6, E70 – E89 |
|  |  |  |

**Supplementary Material**

**SUPPL 1.** ICD-10 codes utilized for study.
